# Pan-immune-inflammation value is a novel prognostic biomarker in pT2-4 gastric cancer

**DOI:** 10.3389/fmed.2026.1802591

**Published:** 2026-03-25

**Authors:** Mingyu Wang, Jiayi Wang, Xinyi Wang, Jian Li, Yingjia Hu, Hongfei Ji, Xingjian Niu, Fujing Wang

**Affiliations:** 1 Department of General Surgery, The Second Affiliated Hospital of Harbin Medical University, Harbin Medical University, Harbin, Heilongjiang, China; 2 Department of Medical Oncology, Harbin Medical University Cancer Hospital, Harbin Medical University, Harbin, Heilongjiang, China; 3 Department of Endocrinology, Health Management Center, Tianjin Union Medical Center, The First Affiliated Hospital of Nankai University, Tianjin, China; 4 Institute of Cancer Prevention and Treatment, Harbin Medical University, Harbin, China

**Keywords:** gastric cancer, pan-immune-inflammation value, prognosis, pT2-4, survival

## Abstract

**Background and objectives:**

Gastric cancer (GC) is a leading cause of cancer-related mortality worldwide and has a poor prognosis. Novel biomarkers are required to predict the survival of patients with this aggressive disease. Although the pan-immune-inflammation value (PIV) has been associated with outcomes in various malignancies, its prognostic role in GC remains unclear.

**Materials and methods:**

Clinical data of 793 patients with pT2-4 GC were retrospectively reviewed and analyzed. An optimal cutoff value was determined using receiver operating characteristic (ROC) curve analysis to stratify patients into high- and low-PIV groups. The prognostic performances of PIV and other immune-inflammatory biomarkers (IIBs) were compared using ROC analysis. Associations between the PIV and clinicopathological characteristics were also assessed. Kaplan–Meier survival curves were constructed for progression-free survival (PFS) and overall survival (OS). Univariate and multivariate Cox regression analyses were performed to determine independent prognostic factors.

**Results:**

The PIV demonstrated superior predictive value for survival compared to other peripheral blood-derived markers. High PIV values were significantly correlated with the *Helicobacter pylori* infection status, a larger tumor size, and an advanced TNM stage. Patients in the high-PIV group, particularly those with stages III and IV disease, had significantly worse PFS and OS. In multivariate analysis, the PIV remained an independent prognostic factor for survival outcomes in patients with pT2-4 GC.

**Conclusion:**

This study established PIV as a significant and independent prognostic biomarker for pT2-4 GC. PIV exhibits a more sensitive predictive ability for the tumor burden and systemic inflammation than other evaluated IIBs, which provides potential clinical value for patients with pT2-4 GC.

## 1 Introduction

Gastric cancer (GC) is a major global health challenge, ranking as the fifth most prevalent malignancy and fourth leading cause of cancer-related mortality worldwide (1). In China, this disease presents a particularly profound clinical challenge, constituting 43.9% of the global GC cases (2). Despite advancements in endoscopic surveillance and molecular diagnostics, GC remains a clinically aggressive disease (3). Approximately 65% of Chinese patients present with advanced (pT2-4) disease at the initial diagnosis, compared to 40%−50% in regions with established screening programs (4). pT2-4 GC is a distinct clinical and biological disease with a significantly poor prognosis and limited curative options (5). Therefore, the identification of appropriate biomarkers is imperative to enhance prognostic evaluation and predict treatment outcomes in pT2-4 GC.

Inflammation and immunity are considered hallmarks of cancer and play crucial roles in tumor development (6). Recently, several immune-inflammatory biomarkers (IIBs) have been evaluated for their prognostic roles in different cancers (7–9). These markers are generally chronic inflammatory markers that are calculated using parameters obtained from circulating elements in peripheral blood specimens, such as the lymphocyte-to-monocyte ratio (LMR) and neutrophil-to-lymphocyte ratio (NLR) (10). As evidence regarding the complex interplay between immunity, inflammation, and cancer has accumulated, composite biomarkers encompassing these elements have emerged as powerful predictors of clinical outcomes in several malignancies (11). Notably, the pan-immune inflammation value (PIV) integrates key inflammatory populations, such as platelets, monocytes, neutrophils, and lymphocytes, and represents a novel biomarker with robust prognostic utility (12–15).

To date, the prognostic role of the PIV in pT2-4 GC has rarely been reported. Here, we retrospectively reviewed the characteristics of patients with pT2-4 GC and investigated the potential role of the PIV as a predictive biomarker in this cohort.

## 2 Materials and methods

### 2.1 Patient selection

This retrospective study included 793 patients diagnosed with pT2-4 GC at the First and Second Affiliated Hospitals of Harbin Medical University between December 2018 and October 2023. An additional 258 samples from healthy individuals obtained from the Health Examination Center of the First Affiliated Hospital were used as normal controls. The inclusion criteria were as follows: (a) pathologically confirmed primary gastric adenocarcinoma and (b) pT stage 2–4 according to the American joint committee on cancer (AJCC) 8th edition (16). The exclusion criteria were as follows: (a) a history of hematological disorders and (b) prior immunomodulatory treatment. The treatment followed multidisciplinary guidelines, encompassing neoadjuvant/adjuvant chemotherapy for resectable disease, palliative systemic therapy for metastatic cases, and surgical interventions for symptom control; 67 patients with HER2 positivity additionally received trastuzumab. All preoperative laboratory data were obtained from an institutional database. Ethical approval was granted by the Ethics Committee of Harbin Medical University, and written informed consent was obtained from all enrolled participants.

### 2.2 Data collection

All medical records were retrospectively reviewed, and clinicopathological data of participants were collected from the patients' records. GC was divided into gastric body, gastric fundus, and gastric antral cancers. The number of blood cells, including lymphocytes, neutrophils, monocytes, and platelets, was assessed using routine blood testing. All laboratory parameters were obtained at baseline (i.e., within one week of the initial diagnosis and before treatment initiation). The PIV was defined according to the formula by Fucà et al. (17): PIV = (neutrophils × platelets × monocytes)/lymphocytes, all measured as 10^9^/L. Additionally, the following ratios were computed: NLR (neutrophils/lymphocytes), systemic immune-inflammation index (SII; [neutrophils × platelets]/lymphocytes), platelet-to-lymphocyte ratio (PLR; platelets/lymphocytes), and monocyte-to-lymphocyte ratio (MLR; monocytes/lymphocytes). Follow-up data were acquired through telephone interviews and institutional records. Accordingly, the progression-free survival (PFS) was defined as the interval from the initiation of treatment to the occurrence of disease progression or death. The overall survival (OS) was defined as the interval from diagnosis to death from any cause, with the last follow-up date serving as the study endpoint.

### 2.3 Statistical analysis

The optimal cutoff values for the PIV and other inflammatory/serum biomarkers were determined using receiver operating characteristic (ROC) curve analysis, with disease recurrence or death as the endpoint of interest. The area under the curve (AUC) with the 95% confidence interval (CI) was calculated for each variable, and the optimal cutoff point was determined using Youden's index (sensitivity + specificity−1). AUC values were classified using AUC thresholds, as follows: 0.9–1.0 (excellent), 0.8–0.89 (considerable), 0.7–0.79 (fair), 0.6–0.69 (poor), and 0.5–0.59 (fail), based on established diagnostic accuracy criteria (18). To assess the stability and optimism of the ROC model, internal validation was performed using bootstrapping with 1,000 resamples.

Associations between PIV groups (high vs. low) and clinicopathological variables were assessed using the Chi-square or Fisher's exact test, as appropriate. The least absolute shrinkage and selection operator (LASSO) regression method was applied to select tumor markers for combination with the PIV. For continuous variables, correlations were evaluated using the Spearman's correlation analysis. Kaplan-Meier curves were constructed to depict survival probabilities, and differences between groups were compared using the log-rank test. Univariate and multivariate Cox proportional hazards regression models were used to identify independent prognostic factors. All statistical analyses were conducted using GraphPad Prism (version 9.0; San Diego, CA, USA) and SPSS (version 20.0; IBM, USA). Two-sided *p*-values < 0.05 were considered statistically significant.

## 3 Results

### 3.1 Patient characteristics

The clinicopathological features of 793 patients with pT2-4 GC enrolled in this study are shown in Table 1. The median age was 57.4 years (range: 23–89 years). This study included 208 females and 585 males. A total of 395 patients had tumors larger than 5 cm in diameter. Among these patients, 73 were classified as pT stage 2, 103 as pT stage 3, and 617 as pT stage 4. The positive expression rates of p53, Ki67, and HER2 immunohistochemical overexpression were 0.779, 0.537, and 0.146, respectively. Other clinical parameters, including the body mass index (BMI), *Helicobacter pylori* (*H. pylori*) infection status, tumor location, and histological features, including differentiation, histological type, and Lauren classification, are presented in Table 1.

**Table 1:** Correlation between PIV and clinicopathological characteristics of pT2-4 GC patients.

**Characteristics**	**Total (*n* = 793)**	**PIV < 303.4 (*n* = 434)**	**PIV≥303.4 (*n* = 359)**	***P*-value**
**Age**, ***y***				
≤ 60	319	172	147	0.707
>60	474	262	212	–
**Gender**				
Male	585	317	268	0.608
Female	208	117	91	–
**Body mass index (BMI)**				
≤ 25	548	299	249	0.886
>25	245	135	110	–
**Helicobacter pylori**				
Negative	198	95	103	**0.028**
Positive	595	339	256	–
**Primary tumor location**				
Proximal	252	145	107	0.540
Middle	76	42	34	–
Distal	465	248	218	–
**Tumor size**				
≤ 5	398	256	142	**< 0.001**
>5	395	178	217	–
**pT stage**				
2	73	54	19	**0.0018**
3	103	58	45	–
4	617	322	295	–
**pN stage**				
0	107	73	34	**0.026**
1	155	81	74	–
2	178	93	85	–
3	353	187	166	–
**pM stage**				
M0	609	342	267	0.141
M1	184	92	92	–
**TNM stage**				
I	52	38	14	**0.004**
II	66	44	22	–
III	489	259	230	–
IV	186	93	93	–
**Differentiation**				
Well	40	21	19	0.957
Intermediate	186	102	84	–
Poor	567	311	256	–
**Histologic type**				
Adenocarcinoma	609	342	267	0.688
Other	184	92	92	–
**Lauren classification**				
Intestinal	341	188	153	0.713
Diffuse	331	177	154	–
Mixed	123	71	52	–
**Ki67**				
>20%	618	341	277	0.633
≤ 20%	175	93	82	–
**P53**				
Positive	426	237	189	0.581
Negative	367	197	170	–
**HER2 IHC**				
0	576	312	264	0.786
1	101	60	41	–
2	61	33	28	–
3	55	29	26	–

### 3.2 Integrated evaluation of the PIV as a predictive and distinguishing immune biomarker in pT2-4 GC

**Figure 1:**
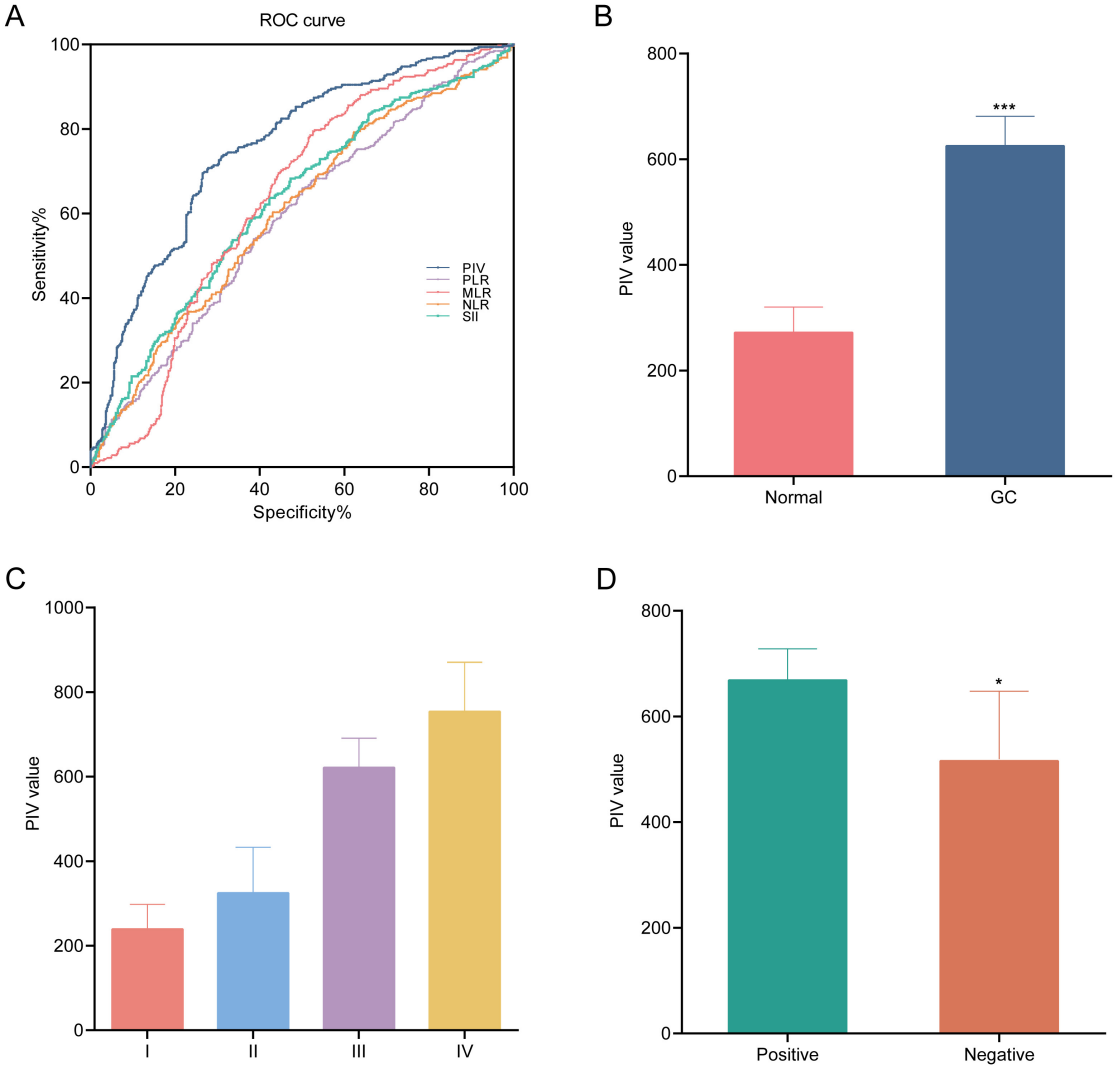
The predictive value of PIV and its association with clinical features in pT2-4 GC. **(A)** ROC curves comparing the prognostic performance of inflammation-related biomarkers. **(B)** PIV levels in pT2-4 GC patients vs normal controls. **(C)** PIV levels across different pT stages. **(D)** PIV levels stratified by *Helicobacter pylori* infection status. ^*^P < 0.05, ^***^P < 0.001

ROC curve analysis was performed to evaluate the predictive performance of inflammation-related biomarkers for pT2-4 GC. The corresponding AUC and optimal cutoff values were determined (Figure 1A). Among all biomarkers assessed, the PIV demonstrated the strongest predictive performance, with an AUC of 0.7558 (*P* < 0.0001) and an optimal cutoff value of 303.4, indicating superior discrimination capability. Although the SII (*AUC* = 0.6272, *P* = 0.020) and MLR (*AUC* = 0.6332, *P* < 0.001) also showed significant predictive values, their overall performance were markedly inferior to that of the PIV. In contrast, the NLR (*AUC* = 0.6037, *P* = 0.187) and PLR (*AUC* = 0.5894, *P* = 0.55) did not exhibit significant discriminative power. Collectively, these findings confirm that the PIV had the most robust predictive efficiency for pT2-4 GC among the investigated biomarkers.

Assessment of the PIV pattern across the clinical groups revealed a distinct profile. The PIV was significantly elevated in patients with pT2-4 GC compared to that in normal controls (Figure 1B). Stratification of patients by the pT stage showed that PIV levels varied significantly across categories and correlated positively with disease progression (Figure 1C). Moreover, the PIV differed significantly between *H. pylori*-positive and -negative patients (Figure 1D). Collectively, these findings suggest that the PIV is a clinically meaningful immunological indicator of pT2-4 GC.

### 3.3 Correlation between the PIV and clinicopathological characteristics of patients with pT2-4 GC

To analyze the clinicopathological relevance, patients were stratified into high- and low-PIV groups based on a predefined cutoff. Among the 793 patients, an elevated PIV was significantly associated with *H. pylori* infection (*P* = 0.028), larger tumor size (*P* < 0.001), advanced pT stage (*P* = 0.0018), pN stage (*P* = 0.026), and TNM stage (*P* = 0.004). No significant between-group differences were observed in age, sex, BMI, histological type, Ki67 expression, or HER2 status.

Correlation analysis revealed a strong positive association between the PIV and CEA (*r* = 0.074, *P* = 0.045), CA125 (*r* = 0.385, *P* < 0.001), AFP (r = 0.101, *P* = 0.022), and negative correlations with Alb (*r* = −0.234, *P* < 0.001). The PIV was not significantly correlated with CA19-9 (Table 2). LASSO regression identified PIV, CEA, CA199, CA125, and AFP as a combined predictive panel (Supplementary Figure 1A), with additional evidence for their prognostic utility provided by ROC curve analysis (Supplementary Figure 1B). These findings collectively underscore the close ties between the PIV and disease aggressiveness and its potential as a composite prognostic biomarker in pT2-4 GC.

**Table 2:** Spearman's correlation coefficient of PIV with clinical parameters.

**Variables**	**Median (range)**	**Number of pairs**	***r***	***P*-value**
CEA	2.39 (0.2–1,000)	734	0.074	**0.045**
CA19-9	12.46	701	−0.014	0.713
CA125	(0.6–1,000)	349	0.385	**< 0.001**
AFP	15.31 (1.87–1,713)	518	0.101	**0.022**
Alb	2.32 (0.5–1000) 36.5 (14.4–51.6)	790	−0.234	**< 0.001**

Bold values mean significant values.

### 3.4 Survival outcomes and subgroup analyses according to PIV values in pT2-4 GC

**Figure 2:**
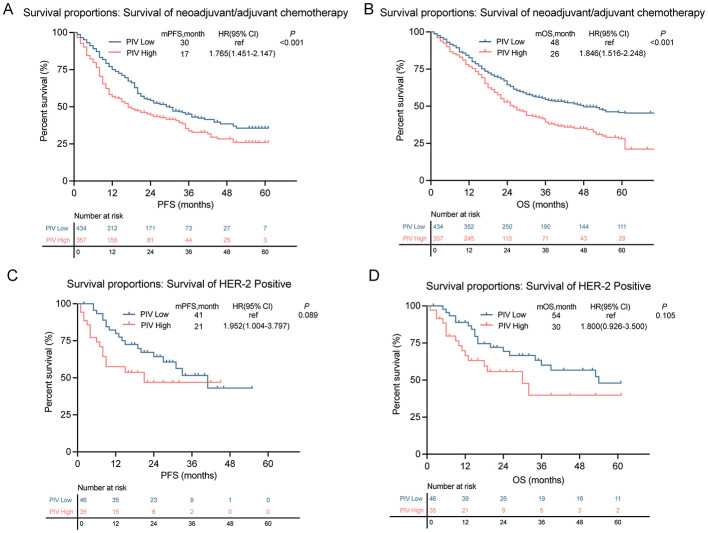
Kaplan-Meier survival curves stratified by high- and low-PIV. **(A, B)** PFS and OS survival rates in patients receiving neoadjuvant/adjuvant chemotherapy. **(C, D)** PFS and OS survival rates in patients treated with or without trastuzumab.

Survival analysis revealed a significant association between an elevated PIV level and poor outcomes. A high PIV level predicted significantly shorter PFS and OS in patients undergoing neoadjuvant or adjuvant chemotherapy (Figures 2A, B). This association was not observed in the trastuzumab-treated subgroup (Figures 2C, D).

**Figure 3:**
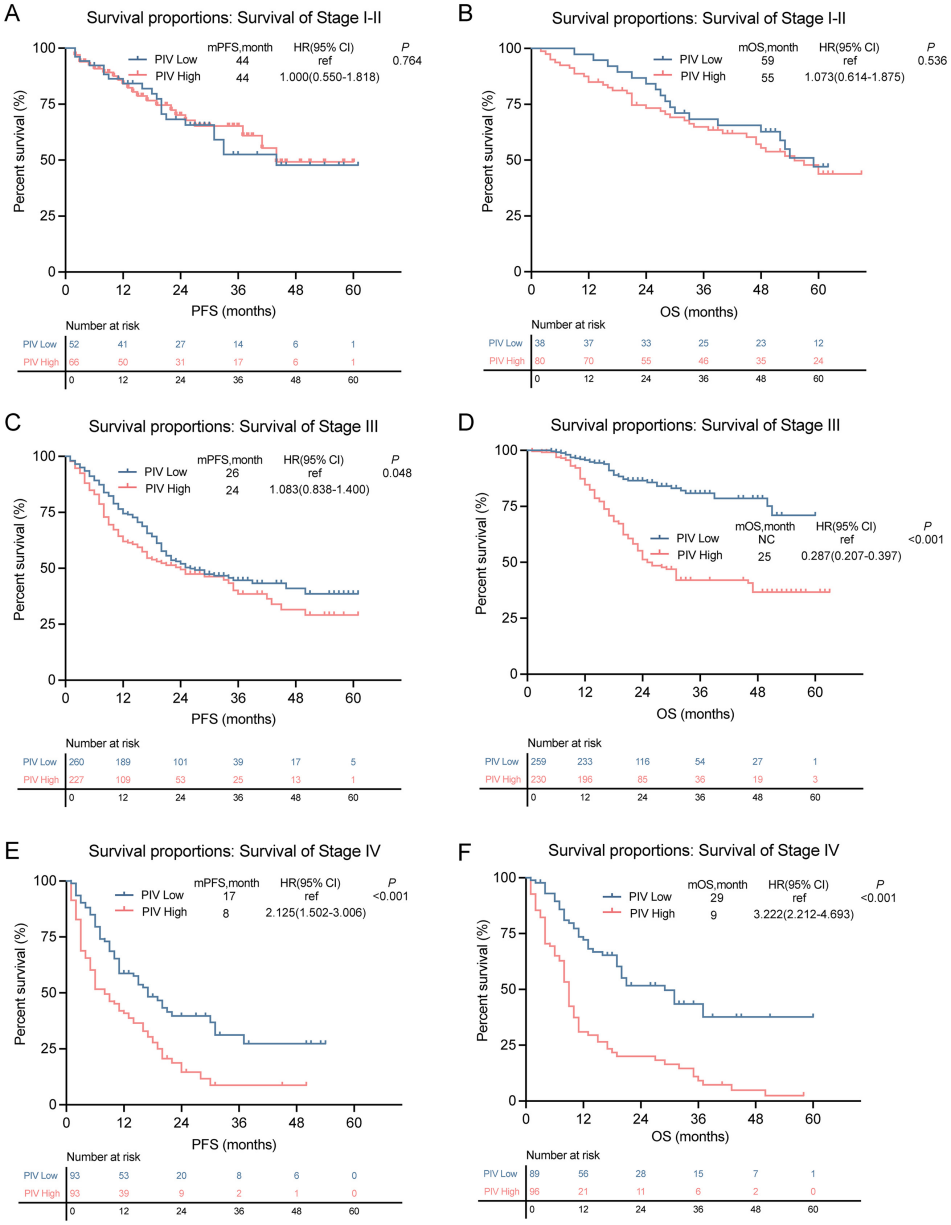
Subgroup survival analysis in TNM stage stratified by high- and low-PIV. **(A, B)** PFS and OS survival rates in stage I–II patients. **(C, D)** PFS and OS survival rates in stage III patients. **(E, F)** PFS and OS survival rates in stage IV patients.

Notably, the prognostic impact of the PIV was modulated by the disease stage. Subgroup analyses found no significant association in early stage (I–II) disease (Figures 3A, B), whereas in patients with late-stage (III–IV) disease, a high PIV level robustly predicted an inferior survival across all endpoints (Figures 3C–F).

Consistent with this stage-specific role, Cox regression analysis confirmed the independence of the PIV. While the univariate analysis highlighted the BMI, TNM stage, and PIV as significant factors for OS, the multivariate analysis confirmed that the PIV was an independent prognostic factor (Table 3). Ultimately, these data substantiate the PIV as an independent prognostic biomarker in pT2-4 GC, providing preliminary exploratory evidence for its application to this patient cohort.

**Table 3:** Univariate and multivariate analysis of prognostic factors for overall survival in pT2-4 GC patients.

**Covariate**	**Univariable**	***P*–value**	**Multivariable**	***P*–value**
	**HR (95%CI)**		**HR (95%CI)**	
Age, y	1.018 (0.840–1.232)	0.859	1.006 (0.816–1.240)	0.958
Gender	0.967 (0.781–1.198)	0.761	0.975 (0.773–1.231)	0.835
Body mass index (BMI)	0.776 (0.631–0.956)	**0.017**	0.863 (0.610–1.221)	0.405
Helicobacter pylori	0.840 (0.679–1.040)	0.110	1.086 (0.238–4.996)	0.915
Primary tumor location	0.990 (0.849–1.153)	0.894	0.981 (0.828–1.163)	0.828
Tumor size	0.846 (0.701–1.021)	0.081	0.788 (0.565–1.099)	0.160
pT stage	1.215 (1.044–1.414)	**0.012**	1.100 (0.770–1.570)	0.600
pN stage	1.095 (1.010–1.187)	**0.028**	1.078 (0.970–1.198)	0.162
pM stage	1.426 (1.147–1.773)	**0.001**	1.342 (0.835–2.156)	0.225
TNM stage	1.245 (1.095–1.415)	**< 0.001**	0.807 (0.527–1.237)	0.325
Differentiation	0.996 (0.845–1.175)	0.964	0.958 (0.805–1.139)	0.624
Histologic type	1.110 (0.741–1.663)	0.612	1.237 (0.808–1.895)	0.327
Lauren classification	0.903 (0.791–1.030)	0.130	0.929 (0.803–1.073)	0.316
Curative vs. palliative treatment	0.998 (0.788–1.264)	0.987	1.327 (0.821–2.144)	0.248
Performance status	1.190 (0.960–1.476)	0.112	1.227 (0.296–5.089)	0.778
Ki67	1.057 (0.874–1.278)	0.567	1.044 (0.850–1.281)	0.683
P53	0.916 (0.759–1.106)	0.362	0.895 (0.731–1.094)	0.279
HER2	1.125 (0.815–1.552)	0.475	1.176 (0.820–1.685)	0.379
PIV	1.901 (1.570–2.301)	**< 0.001**	2.001 (1.584–2.529)	**< 0.001**

Bold values mean significant values.

## 4 Discussion

This retrospective analysis indicated that the PIV is an independent prognostic indicator of survival outcomes in patients with pT2-4 GC. Furthermore, the PIV was significantly associated with key clinicopathological characteristics, including the tumor size and TNM stage, and a high PIV level was associated with poorer PFS and OS in this cohort. These findings suggest that PIV may supplement the prognostic assessment system for pT2-4 GC.

Since pT2-4 GC is an aggressive cancer with a poor prognosis, identifying practical biomarkers to guide perioperative treatment benefits is of great value (19). Inflammation plays a key role in advanced tumor progression (20), and the tumor microenvironment (TME), which is shaped by a complex crosstalk between inflammatory or fibroblastic cells and tumor cells, has long been recognized as a driver of tumor growth (21). Notably, certain immunological elements promote TME remodeling via protumor cytokines and dense stroma formation (22).

Peripheral lymphocytes serve as key mediators of antitumor immunity, reflecting systemic immune activation (23). Systemic and local inflammation profoundly remodel the TME in GC, contributing to a poor prognosis, even with standard treatments (24, 25). Consequently, accessible blood-derived IIBs, including the LMR, NLR, MLR, and PLR indices, have emerged as valuable prognostic tools (26). Compared with traditional IIBs, the newly proposed PIV has demonstrated superior prognostic utility in patients with metastatic colorectal cancer (17). In subsequent studies, the PIV has been confirmed to reflect the prognosis of various malignant tumors, including renal cell carcinoma, breast cancer, lung cancer, renal cell cancer, and prostate cancer (7–9, 13–15, 27–29). When compared with these common IIBs, our findings demonstrated that the PIV outperformed conventional inflammatory indices in predicting survival in pT2-4 GC. This superiority may stem from the ability of the PIV to capture a broader spectrum of immune-inflammatory crosstalk, unlike single-parameter indices, which integrate multiple peripheral blood cell populations (neutrophils, lymphocytes, monocytes, and platelets) that collectively reflect systemic inflammation and immune dysfunction (30). Recent studies have highlighted that such multidimensional inflammatory signatures more accurately mirror the tumor-promoting inflammatory milieu. For instance, a composite inflammatory model that integrated 14 laboratory indices, including platelets, c-reactive protein (CRP), and interleukin (IL)-6, exhibited robust prognostic power in hematological malignancies and supported the superiority of multiparameter scores over single indices (31).

Given its association with aggressive tumor features and poor survival outcomes in pT2-4 GC, we hypothesized that a high PIV level may be a peripheral blood manifestation of the protumor inflammatory state in patients. It is plausible that advanced-stage tumors and larger primary lesions induce sustained systemic inflammation via the secretion of proinflammatory cytokines, which may alter the peripheral blood immune cell composition and thus lead to elevated PIV levels, eventually promoting an immune-evasive phenotype and increasing the tumor burden (32, 33). Our results also identified a significant correlation between the PIV and *H. pylori* infection in patients with pT2-4 GC. Previous studies have demonstrated that *H. pylori* infection drives gastric carcinogenesis via persistent mucosal inflammation and that repeated cycles of inflammation and epithelial damage may contribute to the development of GC (34, 35).

This study had limitations. First, this single-center retrospective exploratory study had sample size constraint and a relatively homogeneous patient population, and lacked an external validation cohort. Second, despite efforts to standardize data collection protocols, heterogeneity in biomarker measurement methodologies could introduce information bias. The PIV is a peripheral blood inflammatory indicator that is easily influenced by the overall condition of the patient, which may affect the stability of the results. Third, incremental prognostic performance metrics, such as C-index improvements, calibration curves, and decision curve analysis, need to be evaluated. Fourth, this study identified clinical correlations between the PIV and the clinicopathological characteristics and survival outcomes; no direct biological experimental data were provided to verify the mechanistic hypotheses. Therefore, the prognostic value and potential clinical application of the PIV in pT2-4 GC requires further verification in multicenter prospective cohort studies, and the underlying biological mechanisms require in-depth exploration.

## 5 Conclusion

Our analysis demonstrates that the PIV is an independent prognostic biomarker in patients with pT2-4 GC. Notably, in the stage III–IV subgroup of this cohort, patients with low PIV levels had significantly superior PFS and OS than those with high PIV levels, and the PIV remained an independent prognostic factor in the multivariate models. Furthermore, these clinical correlation results imply a potential association between systemic inflammation and immune dysregulation and the aggressiveness of pT2-4 GC; however, the specific mechanistic links require further in-depth experimental research.

## Data Availability

The datasets presented in this article are not readily available because all data generated or analyzed during this study are included in this published article. Requests to access the datasets should be directed to niuxingjian@hrbmu.edu.cn.
