# Supplementary figures and images for: Pan-immune-inflammation value is a novel prognostic biomarker in pT2-4 gastric cancer
Source: Front Med (Lausanne). 2026 Mar 25;13:1802591. doi: 10.3389/fmed.2026.1802591 (PMC13056665; doi:10.3389/fmed.2026.1802591)

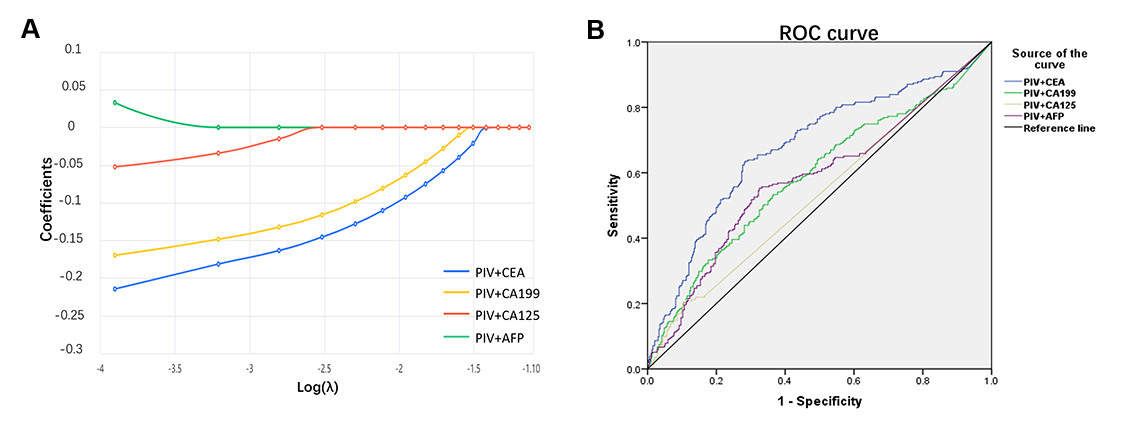

Supplement: Supplementary file 1 [file Image_1.tif]
